# Identification of Enterotype and Its Effects on Intestinal Butyrate Production in Pigs

**DOI:** 10.3390/ani11030730

**Published:** 2021-03-08

**Authors:** E Xu, Hua Yang, Minmin Ren, Yuanxia Wang, Mingfei Xiao, Qingsong Tang, Min Zhu, Yingping Xiao

**Affiliations:** 1 Institute of Animal Nutrition and Feed Science, College of Animal Science, Guizhou University, Guiyang 550025, China; exu@gzu.edu.cn (E.X.); 15761630494@163.com (M.R.); yxwangjya@163.com (Y.W.); L18559501740@163.com (M.X.); tqs5213@foxmail.com (Q.T.); mzhudky@gzu.edu.cn (M.Z.); 2 State Key Laboratory for Managing Biotic and Chemical Threats to the Quality and Safety of Agro-Products, Institute of Agro-Product Safety and Nutrition, Zhejiang Academy of Agricultural Sciences, Hangzhou 310021, China; yanghua@mail.zaas.ac.cn

**Keywords:** Jinhua pig, gut microbiota, enterotype, butyrate

## Abstract

**Simple Summary:**

Enterotype (ET) is defined based on different gut microbial community composition. It has been considered as a critical factor strongly associated with the porcine feed efficiency and growth characteristic. However, little is known about whether the ET clustering depends on the pig breeds and the effects of the different enterotypes on the butyrate metabolism in pigs. Therefore, we studied Jinhua pig’s enterotype by using 16S rRNA high throughput sequencing, and then analyzed the interaction between enterotype and butyrate production. Results showed that we identified three ETs driven by discriminative genera dominated by *Lactobacillus*, *Clostridium sensu stricto 1*, and *Bacteroides*, respectively. Significant differences in the fecal contents of butyrate production and butyrate-producing bacteria were observed among ETs. These findings present a first overview of the enterotypes clustering in Jinhua pigs and provide new insights into the relationship between the different ETs and intestinal butyrate production.

**Abstract:**

Gut microbiota is thought to play a crucial role in nutrient digestion for pigs, especially in processing indigestible polysaccharides in the diets to produce short-chain fatty acids (SCFAs). However, the link between microbiota community structure and phenotypic performances are poorly understood. In the present study, the fecal samples of 105 Jinhua pigs at 105 days of age were clustered into three enterotypes (ETs, ET1, ET2, and ET3) that are subpopulations of distinct bacterial community composition by using 16S rRNA high throughput sequencing. The α-diversity indices (the OTU number and Shannon index) were significantly different among the ETs (*p* < 0.001). At the genus level, the ET1 group was over-represented by *Lactobacillus* (17.49%) and *Clostridium sensu stricto 1* (11.78%), the ET2 group was over-represented by *Clostridium sensu stricto 1* (17.49%) and *Bifidobacterium* (11.78%), and the ET3 group was over-represented by *Bacteroides* (18.17%). Significant differences in the fecal contents of butyrate were observed among ETs, with the highest level detected in ET3 and the lowest in ET2 (*p* < 0.05). Consistently, more copies of the terminal genes for butyrate synthesis, butyrate kinase (Buk) and butyryl coenzyme A (CoA): acetate CoA transferase (But) were detected by qPCR in the fecal samples of the ET3 group as compared to other two groups (*p* < 0.05). In addition, of the two genes, But was demonstrated to be more relevant to the butyrate content (R = 0.7464) than Buk (R = 0.4905) by correlation analysis. In addition, based on the taxonomic analysis, we found that *Faecalibacterium* was the most relevant butyrate-producing genera with fecal butyrate contents in Jinhua pigs, followed by *Butyricicoccus*, *Eubacterium, Butyricimonas, Blautia,* and *Anaerostipes,* all of which showed significantly higher richness in ET3 than as compared to ET1 and ET2 (*p* < 0.05). Collectively, this work presents a first overview of the enterotypes clustering in Jinhua pigs and will help to unravel the functional implications of ETs for the pig’s phenotypic performance and nutrient metabolism.

## 1. Introduction

The pig is an economically important animal. Like humans and other vertebrates, the pig gastrointestinal tract (GIT) harbors a heterogeneous and dynamic ecosystem populated with as many as trillions of commensal microbes, primarily non-pathogenic bacteria [1]. The bacterial population’s collective genome is estimated to contain 3 million genes over 100 times the host genome [2,3]. The gut microbiota is believed to play a central role in the host’s health by harvesting essential nutrients, influencing energy metabolism, maintaining the immune system, and fighting against infection [4,5]. The gut microbial community’s modulations can improve the health and feed efficiency of pigs through microbiota-generated metabolites such as amino acids, vitamins, or short-chain fatty acids (SCFAs) [6]. 

SCFAs, primarily including acetate, propionate, and butyrate, are fermentation products of indigestible carbohydrates by commensal bacteria in the GIT. They are rapidly absorbed and utilized by the host [7,8]. Specifically, the four-carbon SCFA butyrate is the preferred energy source for the colonocytes [9]. In addition, butyrate promotes colonic homeostasis and host health by modulating inflammatory responses, maintaining the intestinal barrier, preventing colonization of enteric pathogens in the GIT, affecting satiety and gut mobility and transit, as well as inhibiting the intestinal cholesterol biosynthesis, diarrhea, and oxidative status [10,11,12]. Butyrate-producing bacteria within the Firmicutes/Clostridium clusters IV and XIVa have been taken as probiotics to increase colonic butyrate levels and optimize gut health [13,14]. Therefore, characterizing, monitoring, and manipulating butyrate production in the pig intestinal tract may benefit animal health and productivity [14].

With the assistance of high-throughput sequencing technology and computational methods that have overcome the limitations of culture-based microbiology, gut microbial diversity in pigs has been described to some extent [15,16]. However, gut microbiota varies largely among individuals on time and space scale [17], which is an obstacle to a better understanding of the complex biological effects of the microbiome. Population stratification is a useful approach to cope with the obstacle, which has found that several equilibrium states—i.e., enterotypes (ETs)—are possible [18]. The term “enterotype” was first raised by Arumugam et al. in 2011, referring to distinct community composition types of human gut microbiota [19]. Three distinct robust clusters driven by discriminative genera were characterized in the human gut microbial community, including *Bacteroides* (ET1), *Prevotella* (ET2), and *Ruminococcus* (ET3) [19]. Since the concept was proposed, ETs have also been detected in several other animals, such as chimpanzees [20], mice [21,22], chickens [23], and even bumblebees [24], proving that ET is generally applicable. With the same methodology, the pig gut microbial composition is structured in two different ET-like clusters [25,26,27]. Ramayo-Caldas et al. found that the ET-like clustering was significantly associated with the pig feed efficiency and growth traits, suggesting the ET concept may have an important role in the pig production industry [26,28]. Although relatively robust, ETs are demonstrated to be strongly associated with the pig age, long-term diets, and even different clustering and grouping strategies [18,28,29,30,31]. However, knowledge is also limited to the effects of the different ETs on the butyrate metabolism in pigs. 

Jinhua pig is one of the most important local breeds in China and is popular for its superior quality pork [32]. In this work, we aimed to identify ETs using the fecal microbiome of 105 Jinhua pigs based on high throughput sequencing and unraveling the relationship between the different ETs and butyrate production. 

## 2. Materials and Methods

### 2.1. Ethical Statement

The experimental animal procedures were approved by the Institutional Animal Care and Use Committee of Zhejiang Academy of Agricultural Sciences (Ethic code: ZAAS-2017-009). 

### 2.2. Animals and Sample Collection

The animals and fecal sample collection were described in our previous report [33]. Briefly, a total of 105 female Jinhua pigs were used in this study. The experimental pig cohort were from 29 Jinhua sows, which were housed in a farrowing house. Pigs were kept in the environmentally controlled facility after weaning, where 5–8 pigs were housed per pen, and raised under the same commercial feed based on corn and soybean meal and management manners. Fresh fecal samples were obtained at 105 days of age and stored at −80 °C until analysis.

### 2.3. DNA Extraction and High Throughput Sequencing 

Genomic DNA was extracted from each fecal sample using the CTAB/SDS method [34]. DNA concentration and purity were estimated by electrophoresis on 1% agarose gels. The V4 region of the bacterial 16S rRNA gene was then amplified using the barcode-fusion forward primer 515F (5′–GTGCCAGCMGCCGCGGTAA–3′) and the reverse primer 806R (5′–GGACTACHVGGGTWTCTAAT–3′). The PCR procedure was run in 30 cycles with an annealing temperature of 50 °C, and the amplicons were purified as previously described [33]. Sequencing libraries were generated using an Illumina TruSeq DNA PCR-Free Library Preparation Kit (Illumina, San Diego, CA, USA) following the manufacturer’s instructions and then sequenced on an Illumina HiSeq platform with 250 bp paired-end reads generated.

### 2.4. Data Analysis

The Sequence data were obtained from the NCBI Sequence Read Archive database with BioProject ID PRJNA412270 (https://www.ncbi.nlm.nih.gov/bioproject/?term=PRJNA412270 (accessed on 1 February 2021)). Cleaned sequencing reads were obtained after removing the primers, barcode sequences, and the low-quality reads from the raw data [35]. Paired-end clean sequence reads were then assembled into tags with the overlapping relationship by QIIME (Quantitative Insights into Microbial Ecology) [36] and assigned to each sample according to the unique bar codes. Tags were then classified into operational taxonomic units (OTUs) at the 97% similarity threshold using USEARCH software [37]. An RDP classifier was used to assign each OTU to a taxonomic affiliation [38]. 

### 2.5. Enterotype Clustering

Enterotype analysis of the pig cohort was done using the genera abundance in each sample as described by Arumugam et al. [19]. In brief, samples were clustered using the probability distribution distance metric related to Jensen–Shannon divergence (JSD) and the Partitioning Around Medoids (PAM) clustering algorithm in the R package “cluster”. The optimal number of clusters was determined using the prediction strength (PS) and silhouette index (SI). Principal-coordinate analysis (PCoA) was performed to visualize these distances using the dudi.pco function in the R package “ade4.” [39]. The number of OTU and the Shannon index calculated using QIIME described the alpha-diversity of each ET [40]. The dominant microbial taxa were statistically analyzed at the phylum and genus levels to show the microbial composition among ETs. 

### 2.6. Physicochemical Characteristics Determination

Procedures set by the Association of Official Analytical Chemists (AOAC, 2006) [41] were used to measure the concentrations of organic matter, total nitrogen, and total phosphorus in all fecal samples. Water concentration was determined as the water lost during the drying of fresh feces at 105 °C until constant weight.

### 2.7. Measurement of Fecal Butyrate 

A total of 100 mg of feces was vortex-mixed vigorously with 1 mL deionized water and centrifuged at 12,000 rpm for 10 min. In addition, 500 μL aliquots of the supernatant were added to 100 μL of 25% (*w*/*v*) mixture of metaphosphoric acid and crotonic acid (internal standard), and then detected by gas chromatography (GC-2010 plus, Shimadzu, Kyoto, Japan) using the method described previously [42]. 

### 2.8. Quantitative PCR (qPCR) Analyses of Key Bacteria and Genes in Butyrate Production

The gene copies of the terminal genes in fecal samples were assessed by qPCR in triplicate on an ABI Prism 7700 Sequence Detector (Applied Biosystems, Foster City, CA, USA) [43,44], including butyrate synthesis, butyrate kinase (Buk), and butyryl CoA: acetate CoA transferase (But) The PCR reaction mixtures contained the extracted DNA as templates, the primer sets listed in Table 1, and SYBR Green PCR Master Mix (Takara, Tokyo, Japan). The thermal cycling conditions were as follows: 95 °C for 2 min and 35 cycles of 15 s at 95 °C, 45 s at 58 °C, and 1 min at 72 °C. Melting curves were plotted to confirm the specificity of the amplification. Quantification was done using standard curves made from known concentrations of plasmid DNA containing the respective amplicon for each set of primers. All qPCR results were expressed as gene copies per g of feces.

### 2.9. Statistics

Statistical analyses and graphing were conducted using SPSS (International Business Machines Corporation, Armonk, NY, USA) statistics software (version 20.0) and Graphpad Prism Program (version 6.0, GraphPad Software Inc., San Diego, CA, USA), respectively. Data are expressed as means ± SEM. The Kruskal–Wallis test was used to compare the α-diversities and the relative abundance of microbial taxa among ETs. The comparison analysis of the butyrate concentrations and butyrate synthesis gene copies (Buk and But) among ETs was performed using the Kruskal–Wallis test [23,27]. Spearman correlation coefficient was used to describe the co-occurrence patterns among the predominant genera and the relationships between relative abundances of butyrate-producing bacteria, fecal contents of butyrate, and gene copies of Buk and But. Significance was set at *p* < 0.05.

## 3. Results

### 3.1. Fecal Microbial Community Composition of Jinhua Pigs

After quality filtering, a total of 8,309,732 DNA sequences were generated from fecal samples of 105 Jinhua pigs, with the sequence number ranging from 46,595 to 98,101 per sample. The sequences were then identified and further clustered into 6903 OTUs at the 97% sequence similarity level. These valid sequences were annotated to represent 40 phyla and 1178 genera by taxonomic analysis using the RDP classifier. At the phylum level, the six most abundant phyla of *Firmicutes*, *Bacteroidetes*, *Actinobacteria*, *Spirochaetes*, *Proteobacteria*, and *Acidobacteria* accounted for more than 90% of the total sequences in most samples. At the genus level, the dominant genera were *Clostridium sensu stricto 1*, *Lactobacillus*, *Streptococcus*, *Terrisporobacter*, *Bifidobacterium*, *Turicibacter*, *Treponema*, *Prevotellaceae NK3B31 group*, *Bacteroides*, *Romboutsia*, *Christensenellaceae R−7 group*, and *Prevotella*. 

### 3.2. Enterotype Clustering and Different Bacterial Community Structures

**Figure 1:**
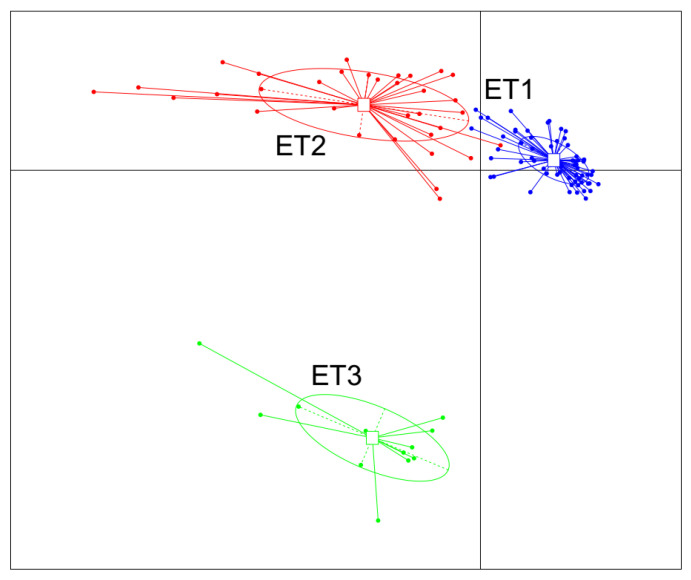
Enterotype clustering in a cohort of 105 experimental Jinhua pigs. The fecal samples were clustered into three distinct ETs using the JSD distance metric based on the relative abundances of bacteria at the genus level. Abbreviation: ET, enterotype.

**Figure 2:**
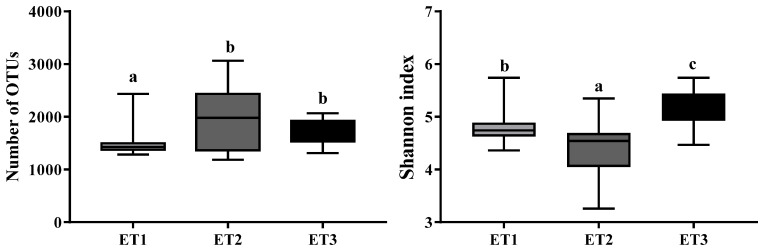
The α-diversity of the three enterotypes. The boxplot shown are means, ranges, and the first and third quartiles. Different letters indicate a significant difference (*p* < 0.05) with the 95% confidence interval. Abbreviation: ET, enterotype.

All 105 samples were clustered into three distinct ETs using JSD distance metric based on the relative abundances of bacteria at the genus level (Figure 1). Microbial complexity in the three ETs was estimated by calculating the α-diversity (Figure 2). The number of OTU and the Shannon index was used to evaluate the community richness and microbiota diversity of each ET group, respectively. The ET2 group had the largest OTU number while the Shannon index of ET3 group was significantly higher than those of the other two groups, indicating that the microbiota in the ET2 group was of higher richness and the bacterial community in the ET3 group was the most diverse. 

**Figure 3:**
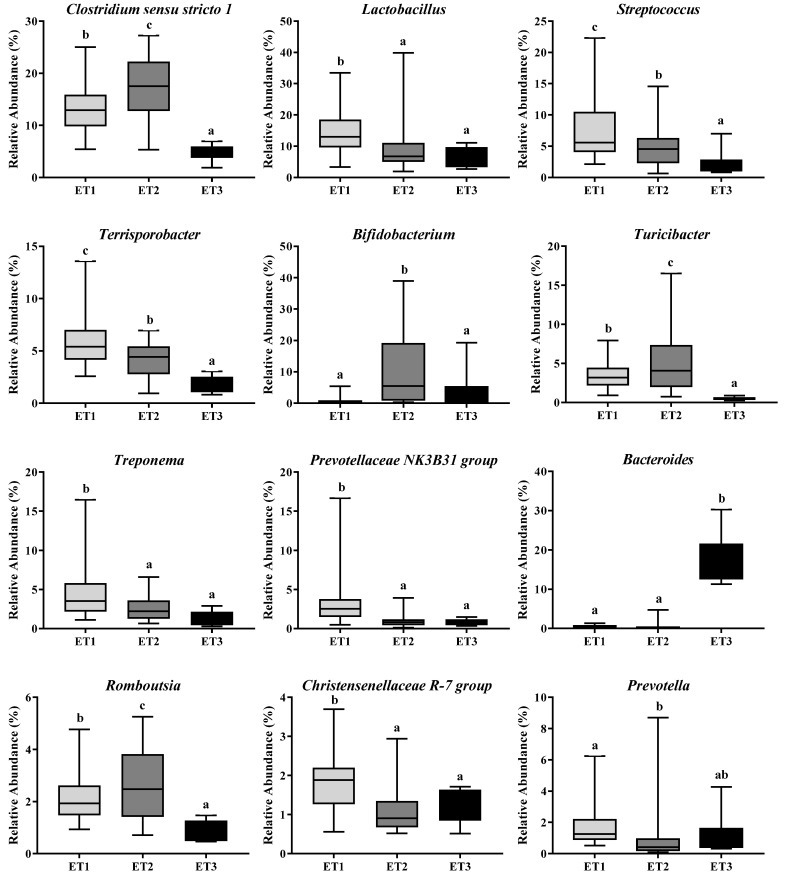
Differentially abundant bacterial genera in three enterotypes. The relative abundances of the 12 most abundant genera in three ETs were illustrated. Different letters indicate a significant difference (*p* < 0.05) with the 95% confidence interval. Abbreviation: ET, enterotype.

**Figure 4:**
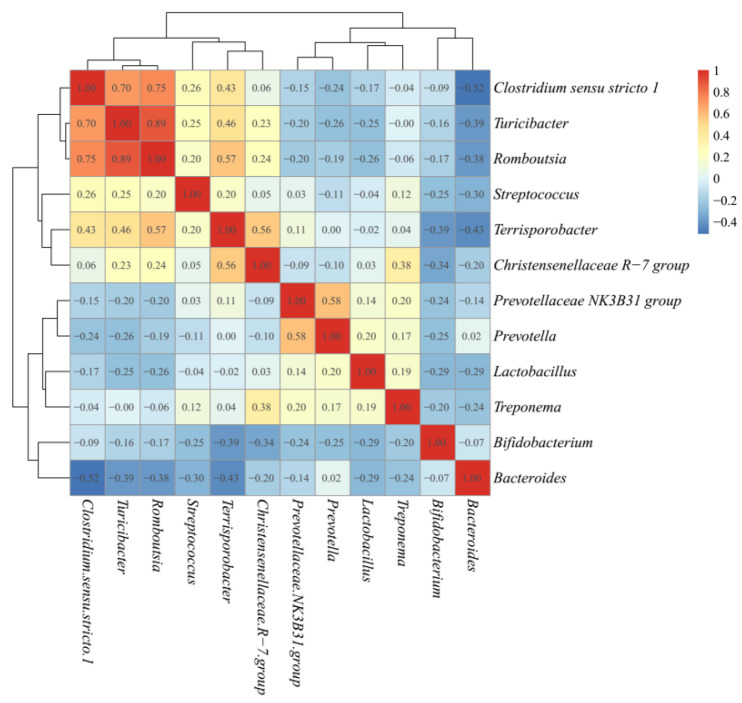
Correlation matrix showing the Spearman’s rank correlations among the most abundant genera. Spearman’s rank correlation coefficients (ρ) range from −1 to 1, corresponding to a strongly positive to a strongly negative correlation, respectively.

The microbiota taxonomic distributions of the three ETs at the phylum and genus levels were analyzed. *Firmicutes*, *Bacteroidetes*, and *Actinobacteria* were the dominant phyla accounting for about 90% of the population in all ETs, but their respective proportions in each ET were somehow different. *Firmicutes* were the most abundant microbes in all three ETs, with even higher richness in ET1 than in the other two ETs (*p* < 0.0001). The relative abundances of *Actinobacteria* in ET2 (*p* < 0.0001) and *Bacteroidetes* in ET3 (*p* < 0.0001) groups were the highest among the three groups, respectively (Table 2). At the genus level, the dominant microbes and their relative abundances varied greatly among ETs (Figure 3). *Lactobacillus* and *Clostridium sensu stricto 1* were the dominant genera in the ET1 group, accounting for 14.39 and 13.41% of the population, respectively; *Clostridium sensu stricto 1* and *Bifidobacterium* were the dominant genera in the ET2 group, accounting for 17.49 and 11.78% of the population, respectively; and *Bacteroides* was the dominant genus in the ET3 group, accounting for 18.17% of the population. Co-occurrence patterns among these genera were determined according to Spearman’s rank correlation (Figure 4). There were strong positive correlations between *Clostridium sensu stricto 1, Turicibacter* and *Romboutsia* (Spearman’s rank correlation coefficients (ρ) were 0.70, 0.75, and 0.89, respectively), while the genuses *Bifidobacterium* and *Bacteroides* were inversely associated with almost every other genus (ρ ranged from −0.07 to −0.52).

### 3.3. Association of Enterotype with Butyrate Production

**Figure 5:**
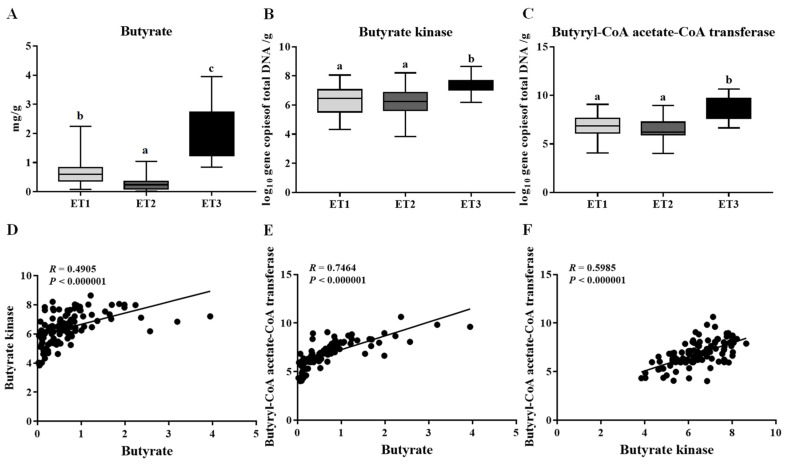
Butyrate contents (**A**) and the gene copies of butyrate kinase (**B**) and butyryl CoA: acetate CoA transferase (**C**) in the fecal samples of three ETs and their correlations as evaluated by Spearman’s rank correlation coefficients (**D**–**F**). The butyrate contents were expressed as mg/g of fresh feces. The abundance of functional genes was expressed as log10 gene copies of total DNA/g of fresh feces. Different letters indicate a significant difference (*p* < 0.05) with the 95% confidence interval. Abbreviation: ET, enterotype.

To assess the tendency of different ETs associating with the pig’s phenotypic performance, we analyzed the physicochemical properties of the fecal samples in the three ETs. However, there was no significant difference in water concentration, pH, organic matter, total nitrogen, and total phosphorus among the three groups (Table 3). The fecal contents of butyrate were further determined and found to be significantly variable across different ETs, with the highest level detected in ET3 and the lowest in ET2 (Figure 5A). Consistent with the higher content of butyrate, more copies of the terminal genes for butyrate synthesis, butyrate kinase (Buk), and butyryl coenzyme A (CoA): acetate CoA transferase (But) were observed in the fecal samples of the ET3 group than those of the other two groups (Figure 5B,C). Correlation analysis confirmed the positive associations between the butyrate contents and the gene copies of Buk and But (Figure 5D,E), wherein the But was much more relevant to the butyrate content than Buk (R = 0.7464 vs. 0.4905).

### 3.4. Butyrate-Producing Bacteria in Different Enterotypes

**Figure 6:**
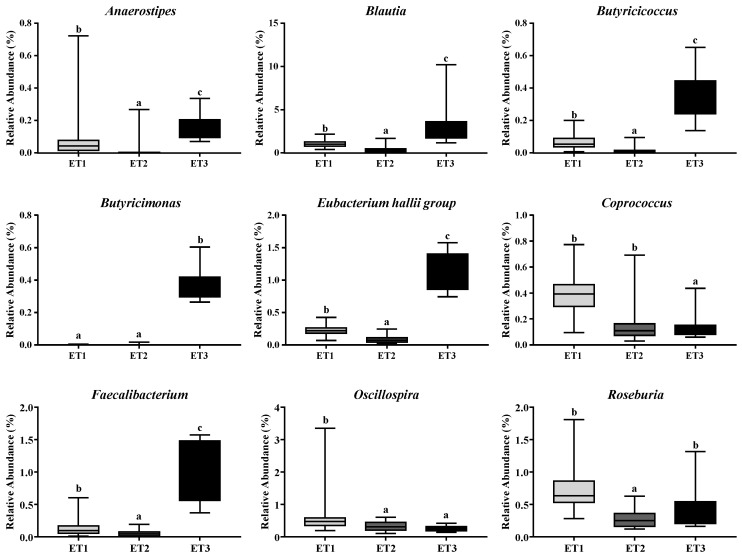
Differentially abundant butyrate-producing bacterial genera in three enterotypes. The relative abundances of the nine most abundant genera related to butyrate production in three ETs were illustrated. Different letters indicate a significant difference (*p* < 0.05) with the 95% confidence interval. Abbreviation: ET, enterotype.

Since the fecal contents of butyrate varied greatly across different ETs, we further identified the butyrate-producing bacteria in the fecal microbiota of the three ETs based on the taxonomic analysis of high throughput sequencing data. The genera, including *Anaerostipes*, *Blautia*, *Butyricicoccus*, *Butyricimonas*, *Eubacterium hallii group*, *Coprococcus*, *Faecalibacterium*, *Oscillospira*, and *Roseburia* that are related to butyrate production, were analyzed. Six out of these nine genera, including *Faecalibacterium*, *Butyricicoccus*, *Eubacterium hallii group*, *Butyricimonas*, *Blautia*, and *Anaerostipes*, showed, in descending order, strong positive correlations with the fecal contents of butyrate as indicated by Spearman’s rank correlation coefficients (Table 4). Notably, all of the six butyrate-producing genera were of significantly higher richness in ET3 than in ET1 and ET2, which might explain the differential butyrate contents in the three ETs (Figure 6). 

## 4. Discussion

The enterotype concept was first raised to stratify the human gut microbiome, which aids to understand and manipulate the complex gut microbiota [19,31]. Since the concept has been extended to other animals, including pigs [20,21,22,23,24,25,26,27]. In the present study, we identified three ETs in 105 experimental Jinhua pigs according to the relative abundance of pivotal bacterial genera in the fecal microbial community characterized by 16S rRNA gene sequencing. *Lactobacillus*, *Clostridium sensu stricto 1,* and *Bacteroides* were the predominant genera in ET1, ET2, and ET3, respectively. Phenotypically, the three subpopulations exhibited a significant difference in colonic butyrate production, probably due to variations in abundance of the butyrate-producing bacteria.

Unlike our present study that divided the pig population into three ETs, most previous research demonstrates that the pig gut microbial composition is structured in two well-defined ETs [25,26,27,28]. However, this classification and the dominant bacterial genera can vary widely with the pig’s ages and breeds. For example, Mach et al. observed a dynamic ET-like clustering shifting across ages in Large White pigs between 14 and 70 days of age and stratified the pigs into two ETs after weaning, primarily distinguished by *Prevotella* and *Ruminococcus* [25]. In addition, Sciellour et al. identified two ETs dominated either by *Lactobacillus* or by *Prevotella*–*Sarcina* in Pietrain pigs at 52 days of age (the post-weaning stage), differing from the two ETs determined from 99 to 154 days (the finishing stage), which were dominated either by *Lactobacillus* or by *Turicibacter*–*Clostridium sensu stricto*, besides a third ET with intermediate genera relative abundance observed at 119 days of age [18]. In this study, the fecal samples were only collected at 105 days of age when the pigs might happen to be in a transition phase. Therefore, the possibility exists that the three ETs in Jinhua pig represent an intermediate state. 

On the other hand, distinct from the rapid-growing lean breeds that were investigated in the previous studies, such as Duroc [27,30], Large White [25], and Pietrain pigs [18], Jinhua pig is a traditional slow-growing breed in China with a high body fat content. It has a significantly different gut microbiome than the lean breeds [32], which might correspondingly lead to the different ET classification. Despite the difference, the *Lactobacillus*-dominant ET1 and *Clostridium sensu stricto 1*-dominant ET2 in Jinhua pigs were very similar to those described in finishing Pietrain pigs [18]. *Bacteroides,* the driving genus in the ET3 group of Jinhua pigs, was also the dominant microbial genus over-represented in the ET1 group of humans and some other animals like mice and broilers [19,22,23]. These similarities suggest some common functional architecture of the microbiota across different pig breeds and even species.

It has been proven that the enterotype-like clustering was significantly associated with pig growth traits [26] and feed efficiency [28], which differently ferment indigestible dietary polysaccharides to produce SCFAs, then absorbed by the host. An important SCFA produced is butyrate that not only being an energy source for the epithelial cells but also influences a wide array of cellular functions affecting colonic health [9,10,11,12]. We then evaluated the relationship between ETs and the pig’s phenotypic performance and found that the fecal butyrate contents were significantly variable across different ETs, with the highest level detected in ET3 and the lowest in ET2. Consistently, ET3 was also more enriched in butyrate synthesis–related genes, Buk and But, than the other two groups. As compared to Buk, But was more closely connected with the butyrate contents according to the correlation analysis, which was in accordance with the previous reports that the last enzymatic step for butyrate formation in the butyrate-producing bacteria in human and pig intestine is commonly But rather than Buk [45,46]. 

The butyrate-producing bacteria in the fecal microbiota of the three ETs were further identified based on the taxonomic analysis of high throughput sequencing data. Butyrigenic bacterial genera *Faecalibacterium* [14], *Butyricicoccus* [25], and *Eubacterium* [14] were most relevant to fecal butyrate contents in Jinhua pigs and exhibited the highest abundance in the ET3 group, explaining the differential butyrate production in the three ETs. Strangely, the genus *Megasphaera* was found to be of very low richness in the fecal samples from Jinhua pigs, in general, the strain of *Megasphaera elsdenii* is the most frequently isolated anaerobe that converts lactate to butyrate in the pig intestine [14]. This may be because our study was based on 16S rRNA high throughput sequencing instead of anaerobic culture-dependent isolation of bacterial colonies that will give preference toward certain bacterial strains. There is also the possibility that the abundance of *Megasphaera* is lower in Jinhua pigs than that in other pig breeds.

## 5. Conclusions

In conclusion, we performed enterotype stratification of the fecal microbiome of 105 Jinhua pigs at 105 days of age by 16S rRNA gene sequencing and computational analysis. We identified three enterotypes driven by discriminative genera dominated by *Lactobacillus*, *Clostridium sensu stricto 1,* and *Bacteroides*, respectively. Significant differences in intestinal butyrate production were observed among different enterotypes. We found that *Faecalibacterium* was the most relevant genera to fecal butyrateate contents in Jinhua pigs, followed by *Butyricicoccus*, *Eubacterium, Butyricimonas, Blautia,* and *Anaerostipes.* Moreover, the gene Butyryl-CoA acetate-CoA transferase was more involved in the terminal enzymatic step for butyrate biogenesis rather than Butyrate kinase. These findings present a first overview of the enterotypes clustering in Jinhua pigs and provide new insights into the relationship between the different enterotypes and intestinal butyrate production, which expanded our understanding of the interactions between gut microbes and pigs.

## Figures and Tables

**Table 1:** Primers used in the present study.

Item	Primers (5′–3′)	Reference
Butyryl-CoA acetate-CoA transferase	fwd AAGGATCTCGGIRTICAYWSIGARATG	[43]
	rev GAGGTCGTCICKRAAITYIGGRTGNGC	
Butyrate kinase	fwd TGCTGTWGTTGGWAGAGGYGGA	[43]
	rev GCAACIGCYTTTTGATTTAATGCATGG	

**Table 2:** Differentially abundant bacterial phyla in three enterotypes.

Phylum	ET1	ET2	ET3	SEM	*p*-Value
Firmicutes (%)	76.5944 ^b^	63.8640 ^a^	60.4627 ^a^	1.1023	<0.0001
Bacteroidetes (%)	14.838 ^b^	8.2120 ^a^	29.3014 ^c^	0.7997	<0.0001
Actinobacteria (%)	1.6287 ^a^	15.5593 ^b^	5.3892 ^a^	1.0396	<0.0001
Spirochaetes (%)	4.5188 ^b^	2.5254 ^a^	1.3508 ^a^	0.2803	0.0001
Proteobacteria (%)	1.1146 ^a^	5.1483 ^b^	2.0045 ^a^	0.2845	<0.0001
Acidobacteria (%)	0.1995 ^a^	1.8077 ^b^	0.0009 ^a^	0.1261	<0.0001

The different superscript letters in the same row represent a significant difference (*p* < 0.05). Abbreviation: ET, enterotype.

**Table 3:** The physicochemical properties of feces in the three enterotypes.

Item	ET1	ET2	ET3	SEM	*p*-Value
Water concentration (%)	72.42	71.18	72.06	2.14	0.746
pH	7.35	7.72	7.06	0.16	0.062
Organic matter (g kg^−1^)	309.42	322.42	289.46	26.18	0.431
Total nitrogen (g kg^−1^)	41.26	45.39	43.64	2.64	0.842
Total phosphorus (g kg^−1^)	31.87	29.35	27.49	1.83	0.262

Water concentration was determined as the water lost during the drying of fresh feces at 105 °C until constant weight. The concentrations of organic matter, total nitrogen, and total phosphorus were expressed as g kg^−1^ of fresh feces. Abbreviation: ET, enterotype.

**Table 4:** Spearman’s rank correlation coefficients between butyrate contents and the gene copies of butyrate kinase and butyryl CoA: acetate CoA transferase.

Genus	Butyrate	Butyrate Kinase	Butyryl-CoA Acetate-CoA Transferase
*Anaerostipes*	0.3047	0.1892	0.1828
*Blautia*	0.5515	0.2678	0.2893
*Butyricicoccus*	0.7134	0.2597	0.4422
*Butyricimonas*	0.6074	0.2709	0.3624
*Eubacterium hallii group*	0.6479	0.3304	0.4268
*Coprococcus*	0.0813	0.1071	0.1353
*Faecalibacterium*	0.8947	0.3404	0.5724
*Oscillospira*	–0.0124	0.0848	0.0944
*Roseburia*	0.0506	0.0201	0.1213

## Data Availability

The Sequence data are available in the NCBI Sequence Read Archive database with accession numbers SRR6109365-SRR6109380 associated with BioProject ID PRJNA412270.

## Abbreviations

AOAC	Association of Official Analytical Chemists
Buk	butyrate kinase
But	butyryl-CoA acetate-CoA transferase
ET	enterotype
GIT	gastrointestinal tract
PCoA	principal-coordinate analysis
SCFA	short-chain fatty acid
